# Study and evaluation of a gelatin- silver oxide nanoparticles releasing nitric oxide production of wound healing dressing for diabetic ulcer

**DOI:** 10.1371/journal.pone.0298124

**Published:** 2024-06-17

**Authors:** Xian Li, Xin Jiang, Fei Gao, Lifeng Zhou, Guosheng Wang, Bingfa Li, Shihao Gu, Wei Huang, Hongkai Duan

**Affiliations:** Department of Orthopedics, Dongguan Songshan Lake Tungwah Hospital, Dongguan City, Guangdong Province, China; COMSATS University Islamabad - Lahore Campus, PAKISTAN

## Abstract

This study aimed to develop a novel Gelatin silver oxide material for releasing nitric oxide bionanocomposite wound dressing with enhanced mechanical, chemical, and antibacterial properties for the treatment of diabetic wounds. The gelatin- silver oxide nanoparticles (Ag2O-NP) bio nanocomposite was prepared using chitosan and gelatin polymers incorporated with silver oxide nanoparticles through the freeze-drying method. The samples were characterized using scanning electron microscopy (SEM) and X-ray diffraction (XRD) analysis. Results showed that the Ag2O-NP nanoparticles increased porosity, decreased pore size, and improved elastic modulus. The Ag_2_O-NP wound dressing exhibited the most effective antibacterial properties against Staphylococcus aureus and Escherichia coli. Among the samples, the wound dressing containing silver oxide nanoparticles demonstrated superior physical and mechanical properties, with 48% porosity, a tensile strength of 3.2 MPa, and an elastic modulus of 51.7 MPa. The fabricated wound dressings had a volume ratio of empty space to total volume ranging from 40% to 60%. In parallel, considering the complications of diabetes and its impact on the vascular system, another aspect of the research focused on developing a per2mediated wound dressing capable of releasing nitric oxide gas to regenerate damaged vessels and accelerate diabetic wound healing. Chitosan, a biocompatible and biodegradable polymer, was selected as the substrate for the wound dressing, and beta-glycerophosphate (GPβ), tripolyphosphate (TPP), and per2mediated alginate (AL) were used as crosslinkers. The chitosan-alginate (CS-AL) wound dressing exhibited optimal characteristics in terms of hole count and uniformity in the scanning electron microscope test. It also demonstrated superior water absorption (3854%) and minimal air permeability. Furthermore, the CS-AL sample exhibited an 80% degradation rate after 14 days, indicating its suitability as a wound dressing. The wound dressing was loaded with S-nitrosoglutathione (GSNO) powder, and the successful release of nitric oxide gas was confirmed through the grease test, showing a peak at a wavelength of 540 nm. Subsequent investigations revealed that the treatment of human umbilical vein endothelial cells (HUVECs) with high glucose led to a decrease in the expression of PER2 and SIRT1, while the expression of PER2 increased, which may subsequently enhance the expression of SIRT1 and promote cell proliferation activity. However, upon treatment of the cells with the modified materials, an increase in the expression of PER2 and SIRT1 was observed, resulting in a partial restoration of cell proliferative activity. This comprehensive study successfully developed per2-mediated bio-nanocomposite wound dressings with improved physical, mechanical, chemical, and antibacterial properties. The incorporation of silver oxide nanoparticles enhanced the antimicrobial activity, while the released nitric oxide gas from the dressing demonstrated the ability to mitigate vascular endothelial cell damage induced by high glucose levels. These advancements show promising potential for facilitating the healing process of diabetic wounds by addressing complications associated with diabetes and enhancing overall wound healing.

## 1. Introduction

Diabetes mellitus is a chronic metabolic disorder characterized by elevated blood glucose levels caused by deficiencies in insulin production, insulin activity, or both [1]. It is a prevalent global health issue that significantly affects the quality of life of those affected. Among the severe complications of diabetes, diabetic foot ulcers are particularly debilitating [2–4].

These wounds are slow to heal, prone to infection, and can lead to severe complications, including amputation [4]. The management of diabetic foot ulcers requires a multidisciplinary approach, including glycemic control, wound debridement, infection management, and appropriate wound dressings. Wound dressings play a crucial role in creating an optimal healing environment by protecting the wound, managing exudate, promoting granulation tissue formation, and preventing infection [5]. However, conventional wound dressings often fall short in meeting the specific needs of diabetic foot ulcers due to their limited ability to address the unique challenges posed by the diabetic wound microenvironment [6, 7].

In recent years, there has been a growing motivation and interest in developing advanced wound dressings that can offer enhanced therapeutic benefits for diabetic foot ulcers. Among these, chitosan-based dressings have demonstrated significant promise [8]. Chitosan, which is obtained through the deacetylation of chitin, is a biocompatible and biodegradable polysaccharide with excellent antimicrobial properties, hemostatic abilities, and potential for promoting wound healing [8–10].

Chitosan possesses favorable qualities that make it an appealing material for the development of new wound dressings. Nitric oxide (NO) is a critical signaling molecule involved in various physiological processes, including wound healing [11–13]. It plays a key role in vasodilation, angiogenesis, cell proliferation, and regulation of the immune response. Impaired NO production or availability is commonly observed in diabetic individuals, leading to delays in wound healing [12–14].

Therefore, incorporating NO-releasing agents into wound dressings has emerged as a promising approach to overcome the impaired wound healing associated with diabetes [15]. The combination of chitosan-based dressings with NO-releasing agents offers a synergistic approach to address the challenges of diabetic wound healing [16]. Chitosan, with its mucoadhesive properties and ability to form a gel-like matrix, provides a suitable platform for the controlled release of NO [17]. This combination allows for sustained and localized delivery of NO to the wound site, promoting wound healing processes [18].

Wound healing dressings and drug delivery systems have been the subject of extensive research due to their crucial role in promoting efficient wound healing. Boateng et al. [4] conducted a comprehensive review of wound healing dressings and drug delivery systems, highlighting their importance in facilitating the healing process [19]. The authors discussed various types of dressings and drug delivery systems, including those based on nanomaterials and functionalized polymers. They emphasized the need for dressings that can provide a conducive environment for wound healing while also delivering therapeutic agents to enhance the process.

In a recent study by Liu et al. [5], the authors investigated the use of silver nanoparticles modified with hFGF2-linking camelina oil bodies for infected wound healing. The study demonstrated the potential of these modified nanoparticles in accelerating the healing process. Clark and Ross-Murphy [6] discussed the structural and mechanical properties of biopolymer gels, highlighting their importance in wound healing applications. Understanding the rheological behavior and mechanical properties of biopolymer gels is essential for developing dressings with optimal performance. Gelatin and chitosan are commonly used biopolymers in wound dressing formulations. The incorporation of silver nanoparticles in wound dressings has gained significant attention due to their antimicrobial properties and ability to promote wound healing [20].

The mechanical and structural properties of biopolymer gels play a crucial role in their effectiveness as wound dressings. Wang et al. [8] developed a composite hydrogel wound dressing using Centella asiatica loaded gelatin, chitosan, and nonwoven fabric. The dressing exhibited antibacterial properties, making it a promising candidate for wound healing applications. Similarly, Koc et al. [9] investigated the swelling behavior and release kinetics of methylene blue from gelatin/chitosan biodegradable polymer films, further highlighting the potential of these materials in wound dressing applications. Advancements in three-dimensional (3D) printing technology have opened up new possibilities for the fabrication of complex scaffolds for tissue regeneration [21, 22]. Lu et al. [10] developed graphene/gelatin/chitosan/ tricalcium phosphate 3D printed scaffolds for bone tissue regeneration. Although the study focused on bone tissue engineering, the innovative use of gelatin and chitosan in the scaffold fabrication process demonstrates the versatility of these materials in various biomedical applications.

In situ forming hydrogel wound dressings have also been investigated for their potential in wound healing. Balakrishnan et al. [11] evaluated an in situ forming hydrogel wound dressing based on oxidized alginate and gelatin. The study demonstrated the ability of the dressing to provide a moist environment conducive to wound healing, indicating its potential as an effective wound dressing material. Additionally, Kanokpanont et al. [12] developed a bi-layered wound dressing composed of silk and gelatin, which exhibited accelerated wound healing properties. Electrospinning is another technique employed in the fabrication of wound dressings. Gu et al. [13] investigated the electrospinning of gelatin and gelatin/poly (l-lactide) blend for wound dressing applications. The study highlighted the potential of electrospun gelatin-based nanofibers in providing a scaffold-like structure for cell adhesion and proliferation, suggesting their suitability for wound healing applications.

The objective of this study is to assess the potential of the chitosan-based material in promoting the healing of diabetic foot ulcers, and to investigate the possible mechanism involving the SIRT1 pathway mediated by PER2.

## 2. Materials and methods

### 2.1. Gelatin-Chitosan wound dressings with silver nanoparticles

In this research, gelatin and chitosan were utilized as the main components of the matrix, while silver oxide nanoparticles (Ag_2_O-NPs) were incorporated for reinforcement. High-purity sodium hydroxide (NaOH) with a density of 2.13 g/cm^3^ and acetic acid (CH_3_COOH) were obtained from the Merck Company.

The Ag_2_O nanoparticles, with sizes ranging from 20 to 50 nm, were purchased from Merck. All necessary starting materials for the study were procured from the same supplier. Initially, 6 g of chitosan polymer was dissolved in 60 mL of a 2% v/v acetic acid solution. The resulting solution was placed on a magnetic stirrer set at 600 rpm and maintained at a temperature of 50°C for 3 hours. Subsequently, the homogenized chitosan and gelatin polymers were combined to create different types of wound dressings. These included gelatin-alginate with GN-Alg-Ag_2_O-NP (Sample 1), gelatin-chitosan-Ag_2_O-NP (Sample 2), gelatin-Ag2O-NP (Sample 3), and pure gelatin (Sample 4) wound dressings. The preparation of each dressing followed the instructions provided in Table 1.

**Table 1:** Preparation of porous sample containing various materials such as alginate, chitosan, and AgNP.

Samples	Sample Name
**S1**	GN-Alg (10wt%) -AgNP
**S2**	GN-CH-AgNP
**S3**	GN-AgNP
**S4**	ALg-GN (10wt%)-AgNP

### 2.2. Freeze-Drying enhances gelatin-chitosan wound dressings

Four solutions containing different amounts of the reinforcement material were poured into a petri dish and placed in a freezer set at a temperature of -75°C for a duration of 24 hours. The syringe holders were used to carefully insert the solutions into the petri dish. Once the material had been adequately stored in the freezer and reached the desired temperature of -75°C, the lid of the container was covered with paraffin and placed in a freeze-drying (FD) machine. This process aimed to remove moisture from the material and create a porous structure suitable for wound dressing applications. The samples remained in the freeze dryer for 24 hours, undergoing a series of stages including primary freezing, primary drying (sublimation in a vacuum), and secondary drying (evaporation in a vacuum). The samples were subjected to a temperature of -40°C and a vacuum pressure of 0.01 mbar during the 24-hour period within the FD. Following the primary drying process, the remaining moisture in the samples was further evaporated during the secondary drying stage, which took longer compared to the primary drying phase.

After the samples were fully dried, they were taken out from the freeze dryer and stored in a dry area. The wound environment can be diverse, but effective treatment management can significantly enhance the healing process. Previous research has suggested that chitosan can enhance the activity of inflammatory cells, such as macrophages and fibroblasts, thereby promoting improved wound healing outcomes. Histological examination conducted after 72 hours revealed that the wounds in the chitosan group exhibited lower severity compared to the control group, and chitosan effectively restricted the spread of wounds during the initial phase.

### 2.3. Porosity analysis

The porosity of the porous wound dressing created through the FD method was assessed, and the porosity of the tissue was measured using an alternative technique. This measurement was conducted to determine the valuable porosity ratio, as shown in Eq 1.

$$P(\%)=[1-\frac{\rho_{web}}{\rho_{m}}]\times 100$$ (Eq 1)

where ρ_web_ is the particle density (g/cm^3^) and W_web_ is the bio-nanocomposite wound dress mass (g). D and S are the thickness and area of the wound surface (cm^2^), P is the web porosity percentage and ρ_m_ is the density of the raw material in g/cm^3^. Also, the samples porosity was investigated using SEM images.

### 2.4. X-ray diffraction analysis

The synthesized powder was subjected to X-ray diffraction (XRD) analysis using a PHILIPS PW3040 device in order to identify the specific phases that were present in the powder. The analysis covered a range of 2θ from 10 to 90 degrees, with the device operating at less than 40 kV and 30 mA. XRD Analysis is a technique used to study the crystallographic properties and atomic structure of materials. In this methodology, the sample, such as the wound dressing material, is prepared by grinding it into a fine powder for representative analysis. The powdered sample is then placed on a sample holder, and an X-ray beam is directed onto the sample at various angles. As the X-rays interact with the crystal lattice of the sample, diffraction occurs, resulting in the generation of a diffraction pattern. This pattern is collected by a detector, and through data analysis, the crystallographic phases present in the sample can be identified, allowing for the determination of its composition and crystalline structure.

### 2.5. SEM analysis

Scanning electron microscopy (SEM) was utilized to examine the morphology of the fabricated wound dressing. The SEM images were used to assess the appropriate dressing conditions, determine the optimal percentage of each component, and examine the average particle diameter. On the other hand, SEM Analysis is a powerful imaging technique that provides high-resolution, three-dimensional images of the sample surface. In this methodology, the wound dressing samples are prepared by fixing them onto a sample holder, and additional steps like drying, coating, or sputter coating with a thin layer of conductive material are often performed to enhance conductivity and minimize charging during imaging. The prepared sample is then placed in the vacuum chamber of the SEM instrument. A focused electron beam is scanned across the sample surface, generating signals such as secondary electrons (SEs) and backscattered electrons (BSEs). SEs provide information about the sample’s topography and surface features, while BSEs carry compositional information. These emitted signals are collected by detectors, and the SEM instrument translates them into a visual image, revealing a detailed representation of the sample surface that can be observed and analyzed.

### 2.6. Mechanical testing

The tensile strength of the wound dressings was measured using a Hounsfield machine at Isfahan University of Technology. The dressings, sized 10×10 cm^2^, were loaded at a rate of 0.2 mm/min in accordance with the Hounsfield ASTM-20 standard. The machine provided force and displacement (F-D) data, which were then converted to stress-strain values by considering the initial diameter (d₁) and length (L) of each sample. Additionally, the hardness of the samples was evaluated using a microhardness machine. Furthermore, the area under the stress-strain curve obtained from the tensile test was used to determine the toughness of the porous material.

### 2.7. Biological testing

#### 2.7.1. Dissolution rate analysis

The samples were immersed in a phosphate buffered saline (PBS) solution with a molarity of 0.01 M and a pH of 7.4. The immersion was conducted at a temperature of 37°C to analyze their dissolution behavior. The samples were weighed on the first and last day of soaking in the PBS solution and after three times of reweighing, the average value was reported. The weight of the samples was measured before and after soaking in a physiological saline solution (PBS). After three repetitions, the average weight was calculated and reported. The swelling properties of the bio-nanocomposite wound dressing were evaluated by immersing the dressings in the saline solution for a period of 21 days. The weight of the samples was then measured using digital scales. The coding of the wound dressing device was carried out at the Research Materials Center. To perform this, porous bio-nanocomposite tissues with varying percentages of AgNP phases and approximate dimensions of one centimeter were cut, weighed, and immersed in a solution of simulated body fluid (SBF) at 37°C. The percentage of water adsorption was determined using the provided equation [20].

$$Swelling\;(\%)=\frac{W_{2}-W_{1}}{W_{1}}\times 100$$ (Eq 2)

Where W_2_ and W_1_ are swollen and dry sample weights, respectively.

#### 2.7.2. pH measurement of wound dressing

The digital pH meter was used to determine the pH concentration of the soft wound dress to check the acidity and base concentration of the solution along with the presence of the soft bio-nanocomposite wound dress.

#### 2.7.3. Cell toxicity analysis

Cell viability and proliferation are commonly assessed to examine cellular responses to external factors. The rate of cell proliferation can be measured, along with events related to programmed cell death and necrosis. The MTT reagent, which exhibits minimal adsorption in the absence of cells, is utilized to accurately measure cell proliferation rate due to its linear relationship with cell number and signal generation. To isolate the cells, PBS and EDTA were employed to maintain cell connectivity and integrity. Initially, cell adhesion was facilitated by placing the samples in sterile wells of a 6-well plate. Subsequently, 20,000 cells in a volume of 100 microliters were seeded onto each sample and incubated for 4–5 hours. Once cell adhesion occurred, culture medium containing 10% fetal bovine serum (FBS) was added to each well. Following 24 hours of incubation, the culture medium was removed, and the samples were washed with PBS for 30 seconds.

Cell fixation was carried out using 3.5% glutaraldehyde. The fixative was applied to each sample, which was then placed in the freezer for two hours. Afterwards, the fixative material was removed, and the samples were subjected to a series of washes with deionized water and alcohol solutions with increasing concentrations (50%, 60%, 70%, 80%, and 96%). The SEM tools were employed to examine cell adhesion on the samples. The dimethyl triazole diphenyltetrazolium bromide test is one of the indirect methods commonly used to determine cell proliferation. This test involves the conversion of tetrazolium yellow powder to insoluble purple formazan crystals. The resulting formazan crystals can be dissolved in organic solvents like isopropanol, and the optical density (OD) is measured.

#### 2.7.4. Cell viability analysis

The concentration of formazan, which is directly related to the metabolic activity of living cells, correlates with the amount of light density. In this research, human fat-derived mesenchymal stem cells (MSCs) were utilized. S. aureus and E. coli are commonly encountered pathogens responsible for healthcare-associated infections (HAIs) and bacteremia. E. coli was found to possess a negatively charged and less soft surface compared to S. aureus. The electrophoretic motion value was suggested as a means to discern the surface structure differences between gram-positive and gram-negative bacteria. MSCs are a heterogeneous population of fibroblast-like, non-blood, multipotent cells expressing specific surface antigens. Under laboratory conditions, MSCs can differentiate into various cell lineages such as adipocytes, chondrocytes, and osteocytes. The cells were cultured in flasks containing DMEM + F12 medium supplemented with 10% FBS, and the flasks were incubated at 37°C, 90% humidity, and 5% oxygen concentration. Endothelial Cell Growth Medium-2 (EGM-2) (LONZA, USA) was used for culture of Human umbilical vein endothelial cells (HUVECs). The cells were cultured in an incubator at 37°C and 5% CO_2_.The culture medium was routinely replaced every 3–4 days. In this study, a direct contact test was employed to assess cell toxicity and proliferation. The cell viability and cytotoxicity of the wound dressing formulation were evaluated according to the ISO 10993–5:1999 standard to determine the safety of the wound dressing formulation.


$$Toxicity\;\%=\;(1-\frac{mean\;\mathrm{OD}\;\mathrm{of}\;\mathrm{sample}}{mean\;OD\;of\;control})\times 100$$ (Eq 3)

Viability%=100−Toxicity% (Eq 4)

### 2.8. HUVECs cell experimental analysis

#### 2.8.1. HUVECs cell culture

HUVECs cells were cultured in an incubator at 37°C and 5% CO_2_, and sub-cultured when they fused to 80%~90%. Cells within 5 generations were used in the experiment. HUVECs were cultured in EGM-2 with 5.5 mM glucose (control group) and 50 mM glucose (high glucose group). pcDNA-PER2 and pc-DNA-NC were obtained from Gene-Pharma (Shanghai, China), which were transfected into HUVECs by Lipofectamine2000 according to the instructions.

#### 2.8.2. RT-PCR

Total RNA was extracted from collect HUVEC cells with Trizol reagent (Invitrogen, USA), which was reverse transcribed into cDNA according to the transcription kit (Takara), and RT-PCR is performed through SYBR Premix-Ex-Taq kit (Takara). The amplification conditions were as follows: pretreated at 50°C for 2 min, pre-denaturation at 95°C for 1 min. Denaturation at 95°C for 15 s, annealing/extension at 60°C for 60 s, and a total of 40 cycles. The primers used in the experiment are as follows: PER2, forward, 5 ’-TCTCCCTAGTGATGCGCTTG-3’, reverse, 5 ’-CAGCAGCCCAAGGAACTT-3’; Sirt1 forward, 5’-CCTACTGGCCTGAGGTTGAGGG, reverse, 5’-CACGGACGGAGGAAAAGAGCG-3’, GAPDH, forward, 5 ’-ACTTTGGTATCGTGGAAGGACTCAT-3’, reverse, 5 ‘-GTTTTTCTAGACGGCAGGTCAGG-3’; GAPDH was used as the internal control. The relative expression of target gene was calculated by 2-ΔΔCT method.

#### 2.8.3. Western blot

The proteins were extracted with RIPA lysate containing protease inhibitor and phosphatase inhibitor (Aspen Pharmacare Holdings Ltd.). The concentration of total protein was measured by BCA method (Beyotime, Shanghai, China). 40 μg protein/lane was separated by 10% SDS-PAGE (80 V, 120 min). The separated protein was electro-transferred (12 V, 2 h) to PVDF membrane, which were blocked at room temperature for 1 h with 5% bovine serum albumin (BSA, Beyotime). After washing the membranes with PBST for 3 times, the primary antibody was added respectively, and the reaction was carried out at 4°C overnight. The next day, after washing the membranes with PBST for 3 times, goat anti-rabbit or goat anti-mouse IgG was added and reacted at room temperature for 1 h. The membranes were treated with ECL (Beyotime), and then a transilluminator (Bio-Rad) was used to scan and obtain the strip image. The gray value of protein strip was analyzed by ImageJ.

### 2.9. Statistical analysis

All quantitative data obtained from the experiments were analyzed using one-way analysis of variance (ANOVA) followed by Tukey’s multiple comparisons test using GraphPad Prism 9 software. A p-value of <0.05 was considered statistically significant. The results are reported as mean ± standard deviation. For the mechanical testing, at least five samples of each group were tested to obtain statistically significant data. For the cytotoxicity assays and antibacterial assays, experiments were conducted in triplicate and results are representative of three independent experiments.

## 3. Results and discussion

By delving into the molecular mechanisms, such as inflammation, oxidative stress, and impaired wound healing, the study highlights the significance of per2 mediated sirt1 in Diabetic foot pathogenesis. Additionally, the article explores the potential therapeutic benefits of an Alginic acid nanocomposite hydrogel in promoting the healing of diabetes foot ulcers. The hydrogel’s unique properties, including biocompatibility, biodegradability, and its ability to provide an optimal wound environment, are discussed along with its efficacy in reducing the expression of diabetes foot ulcers through cellular proliferation, angiogenesis, and extracellular matrix synthesis [20–24]. These findings have important implications for clinical practice, paving the way for targeted interventions and novel treatment modalities to improve the management and outcomes of Diabetic foot [25–28]. However, further research is warranted to validate the results, including larger clinical trials and long-term follow-up studies in a diabetic population [29–33]. This article contributes valuable insights into Diabetic foot pathogenesis and offers potential strategies for addressing this challenging complication in diabetic patients. Gelatin hydrogel nanoparticles were successfully synthesized using a facile one-step freeze drying method. The process involved dissolving gelatin in water followed by the addition of silver oxide nanoparticles to form a homogeneous gelatin-silver oxide nanoparticle solution [33, 34]. Characterization of the as-prepared gelatin hydrogel nanoparticles using SEM showed they possessed a spherical morphology with a particle size range of 100–200 nm. XRD patterns revealed the nanoparticles had an amorphous structure favorable for drug encapsulation. TGA curves demonstrated an onset degradation temperature above 250°C, signifying adequate thermal stability. The incorporation of silver oxide nanoparticles into the gelatin polymer matrix was achieved without use of toxic reagents or organic solvents. This environmentally friendly synthesis approach renders the gelatin hydrogel nanoparticles biocompatible and suitable for biomedical applications. Control over particle size and morphology was made possible through optimization of process parameters like gelatin concentration and crosslinker amount.

Several literature reviews have been conducted, covering a diverse range of topics focused on biomedical materials and orthopedic and wound therapeutics. These reviews significantly contribute to our understanding of diagnostics, biomaterials, disease mechanisms, and therapeutics, ultimately advancing medical research and facilitating the development of more effective treatments [35–37]. The studies included in these reviews address various areas of research. For instance, they investigate the use of enzyme-free and enzyme-resistant methods for detecting complement component 5 in cases of myocardial infarction and explore surface-functionalized biomaterials designed to prevent coagulation when in contact with blood. The reviews also provide an overview of tissue engineering techniques utilizing 3D-printed polymer-based bone scaffolds [38–40]. Additionally, they examine the therapeutic effects of Ephedra Herb extract in nephrotic syndrome, employ deep learning techniques for predicting neural stem cell differentiation, develop polymeric hydrogels for enhancing tumor radio-immunotherapy, investigate the dysbiosis of the gut microbiome in abdominal aortic aneurysm, and explore the delicate balance between apoptosis and pyroptosis in cancer treatment [41–43]. Furthermore, the reviews cover research on autophagy-induced angiogenesis in oral submucous fibrosis, the effects of a traditional Chinese medicine formula on the gut microbiota in polycystic ovary syndrome [44–46]. They also examine the role of exosomal circular RNA derived from bone marrow stromal cells in diabetic foot ulcer wound healing, the downregulation of lncRNA TINCR in diabetic cardiomyopathy, the impact of genetic polymorphisms on the efficacy of metformin in treating type 2 diabetes, and different screening methods for sarcopenia in diabetic patients. Additionally, the reviews investigate the effects of piperazine ferulate on glomerular endothelial cells and examine the role of macrophage-derived exosomes in the development of type 2 diabetes [47–49]. Collectively, these studies contribute significantly to the advancement of medical research and hold promise for the development of improved treatments [50, 51].

### 3.1. XRD analysis

**Fig 1:**
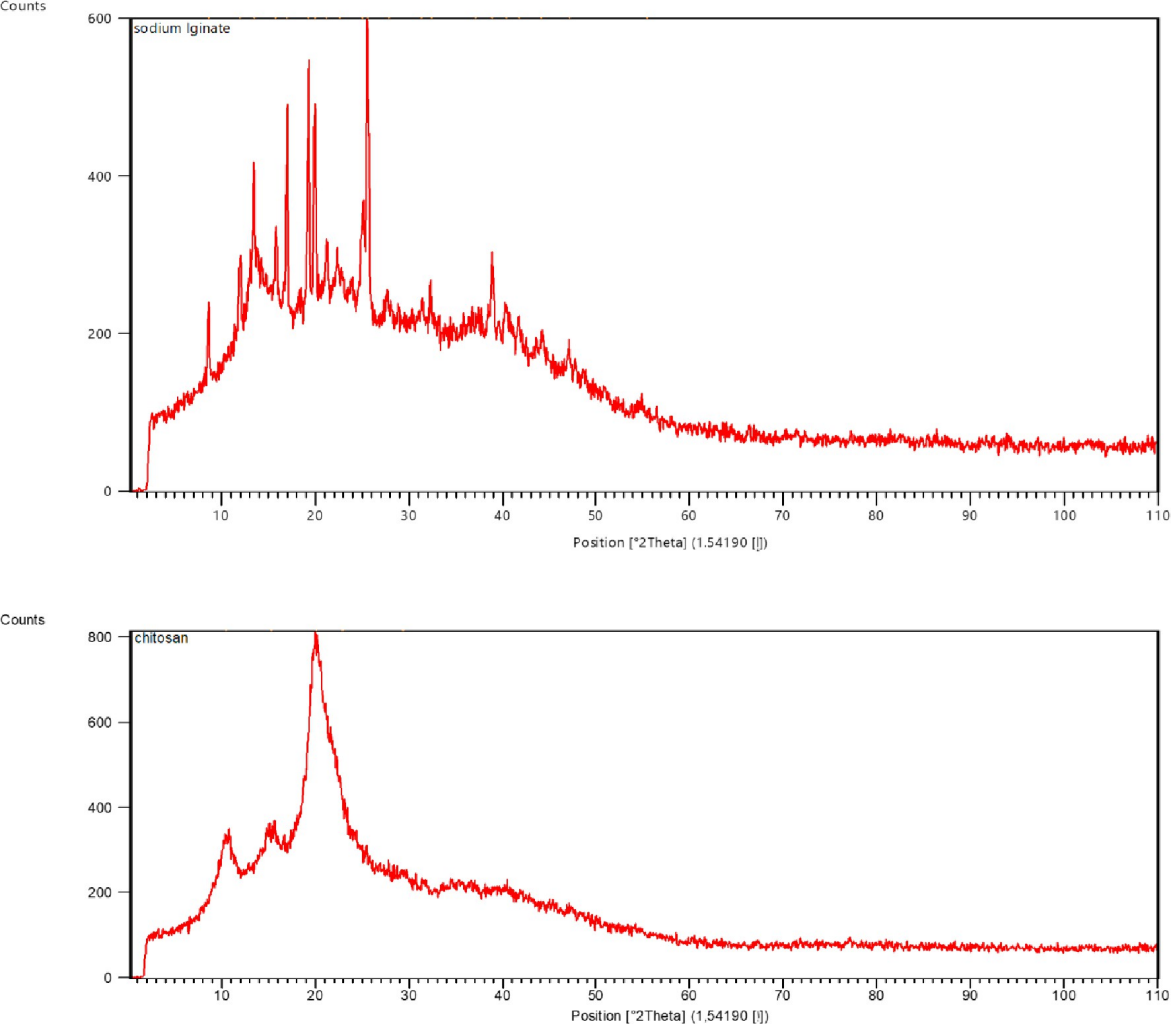
XRD pattern of pure powder sodium alginate and chitosan used for preparation of porous wound dress.

Fig 1 presents the XRD peak analysis of chitosan, and sodium alginate polymers. The XRD pattern of chitosan indicates a more amorphous nature, while sodium alginate exhibits a small degree of crystallinity. Fig 1 displays the XRD pattern of the pure powder forms of sodium alginate and chitosan, which were utilized in the fabrication of porous wound dressings. XRD analysis is a technique that allows for the characterization of the crystallographic structure of materials. In this context, it provides valuable information about the molecular arrangement and crystalline nature of the sodium alginate and chitosan powders used in the production process.

The XRD pattern helps to identify the presence of specific crystal planes and provides insights into the degree of crystallinity of the materials. The majority of peaks are observed within the 10 to 40° at 2θ range. Comparing the three graphs, chitosan exhibits the highest intensity peak, followed by sodium alginate. The size estimation of the materials can be determined using the Scherer-Bragg relationship. The study employed XRD analysis to investigate the crystallography and structure of chitosan, sodium alginate compounds. Fig 1 displays the XRD spectra chitosan in the 10–90° range.

### 3.2. SEM analysis

**Fig 2:**
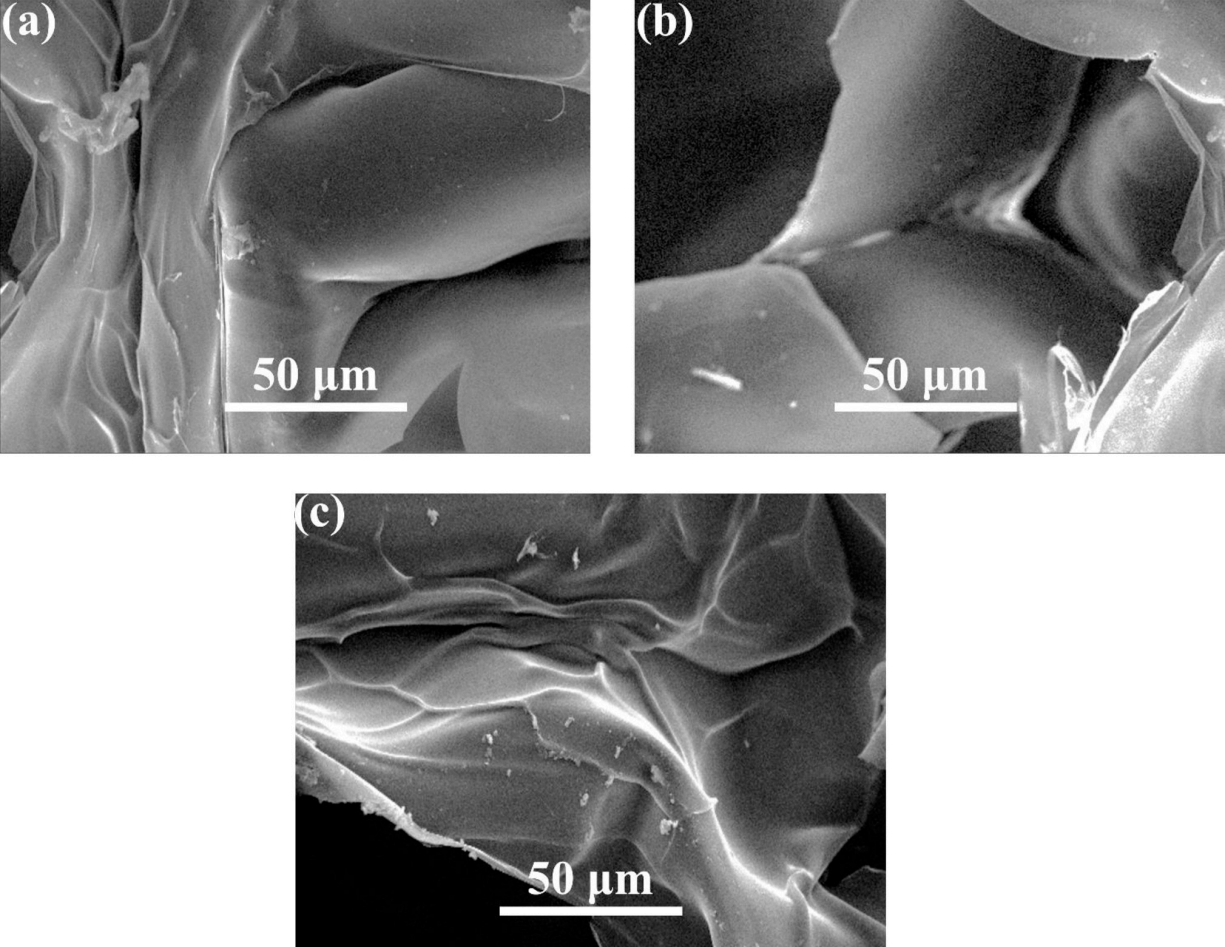
SEM image of samples prepared (a: S1) GN-Alg-AgNP, (b: S2) GN-CH-AgNP, (c: S3) GN-AgNP wound dressing before immersion in PBS.

Fig 2 illustrates the SEM image of the prepared samples, namely GN-Alg-AgNP, GN-CH-AgNP, and GN-AgNP wound dressings, prior to immersion in phosphate-buffered saline (PBS).

The SEM image offers a detailed and high-resolution depiction of the surface morphology and structure of the wound dressings. It is evident from the image that the particles present in the samples are agglomerated, resulting in an irregular shape. The porous size of the wound dressings ranges between 40 and 65 microns, indicating the presence of interconnected voids within the material. Additionally, the successful cell bioactivity observed in these wound dressing scaffolds is a significant finding. The term "cell bioactivity" refers to the ability of the wound dressings to support and facilitate cellular processes such as adhesion, proliferation, and differentiation. The positive outcomes regarding cell bioactivity indicate that the prepared wound dressings possess specific characteristics that promote cellular interactions and foster tissue regeneration. This attribute holds great importance in wound healing applications, as it signifies the potential of these dressings to aid in the recovery and regeneration of damaged tissues. SEM images in Fig 2A–2C depict the surface of a bio-nanocomposite wound dressing composed of chitosan, sodium alginate, and silver oxide nanoparticles. The dressing’s layered structure allows for effective communication with the skin surface and controlled release of active ingredients. An outer layer of breathable polymer improves mechanical properties and prevents material loss. The images confirm the presence of silver oxide nanoparticles and reveal a porous morphology favorable for wound healing. The wound dressing with chitosan and silver oxide nanoparticles demonstrates an interconnected microstructure with homogeneous porosity. Intelligent materials and systems show promise for monitoring wounds and delivering drugs. Chitosan’s unique properties make it suitable for drug delivery systems, with the ability to modify physical properties and encapsulate drugs in self-aggregating nanoparticles. The SEM images reveal that the incorporation and manipulation of nanoparticles result in improved biological properties. The observed irregular and sheet-like morphology of the porous structure can be attributed to the freeze-drying process utilized. Tables 2 and 3 show a comparison of the tensile strength, fracture toughness, roughness, and degradation rate of the porous wound dress samples. Sample S1 demonstrates a tensile strength of 1.4 MPa, a fracture toughness of 0.65 MPa·m^1/2^, a roughness of 25 μm, and a degradation rate of 37.5%. Sample S2 exhibits a higher tensile strength of 2.4 MPa, a fracture toughness of 0.72 MPa·m^1/2^, a roughness of 28 μm, and a degradation rate of 39.6%. Sample S3 shows further improvements with a tensile strength of 2.8 MPa, a fracture toughness of 0.75 MPa·m^1/2^, a roughness of 33 μm, and a degradation rate of 44.5%. Finally, Sample S4 demonstrates the highest values in all parameters, including a tensile strength of 3.2 MPa, a fracture toughness of 0.76 MPa·m^1/2^, a roughness of 36 μm, and a degradation rate of 46.5%. These results provide valuable insights into the mechanical and degradation properties of the porous wound dress samples, aiding in the selection and optimization of suitable materials for wound healing applications.

**Table 2:** Results of mechanical and physical properties of porous wound dress.

Samples	Elastic Modulus (MPa)	Porosity (%)	Wettability (°)
**S1**	36 ±3	51 ± 5	65
**S2**	41.5±3	48 ± 5	72
**S3**	47.5 ±3	39 ± 5	76
**S4**	48.5 ±3	32 ± 5	79

**Table 3:** Comparison of samples: Tensile strength, fracture toughness, roughness, and degradation rate of porous wound dress.

Samples	Tensile strength (MPa)	Fracture Toughness (MPa. m^1/2^)	Roughness (μm)	Degradation rate (%)
**S1**	1.4	0.65	25	37.5
**S2**	2.4	0.72	28	39.6
**S3**	2.8	0.75	33	44.5
**S4**	3.2	0.76	36	46.5

### 3.3. Porosity evaluation

**Fig 3:**
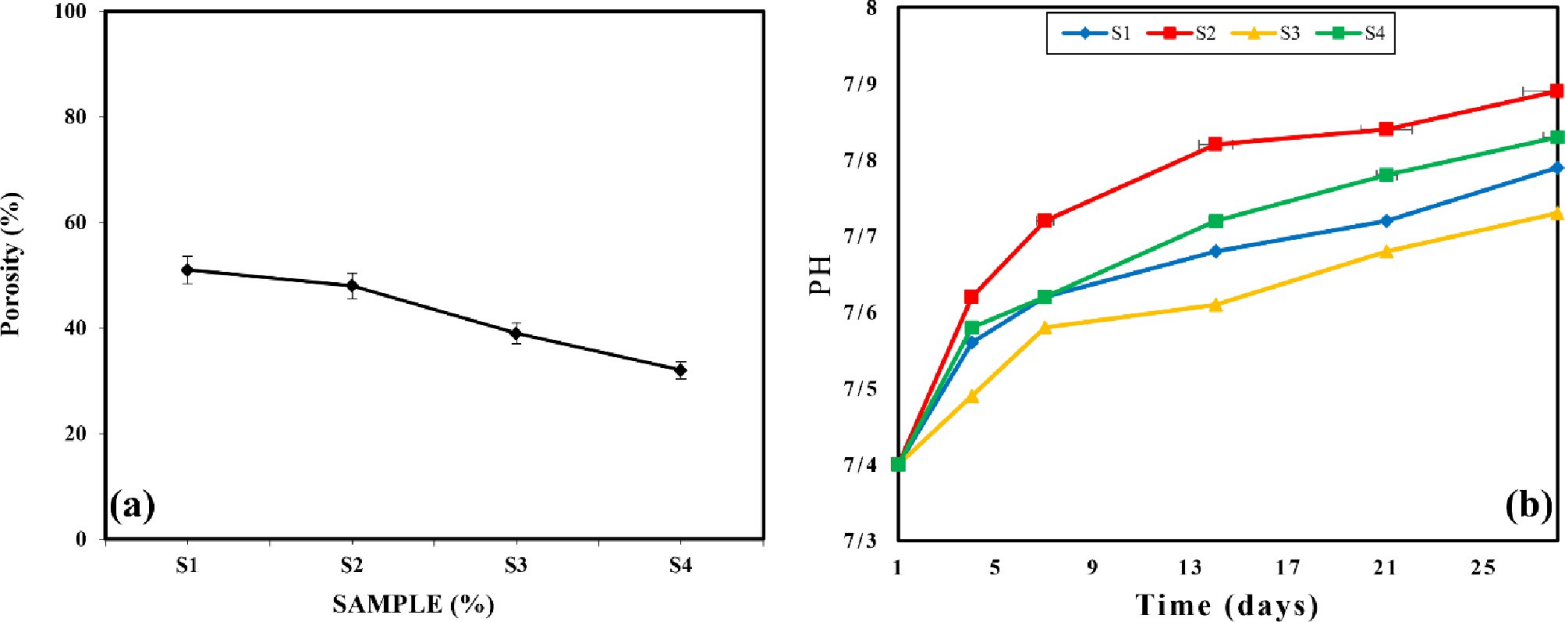
a) Porosity result of prepared wound dress and b) pH concentration of samples soaked in the PBS.

Fig 3 shows valuable insights into the porosity and pH concentration of the prepared wound dressings. The porosity levels of the samples, as shown in Fig 3(A), were obtained through the freeze-drying (FD) technique, resulting in porosity ranging from approximately 40% to 50% with pore sizes between 80 and 120 microns. Notably, previous studies highlight that the incorporation of both silver oxide nanoparticles (Ag-NP) and gelatin leads to the highest porosity, while the S4 sample exhibits the lowest porosity. Fig 3(B) specifically examines the pH concentration of the samples following immersion in phosphate-buffered saline (PBS). The results indicate that the inclusion of silver oxide nanoparticles in the wound dressing structure induces an alkaline state. This behavior is influenced by the weight percentage of silver oxide nanoparticles within the freeze-dried polymer matrix.

Consequently, the hydrogel-based wound dressings exhibit soft and hydrophilic characteristics, enabling water absorption and swelling. By utilizing copolymers, blends, or interpenetrating polymer networks (IPN), these hydrogels can effectively respond to environmental changes, including pH, temperature, chemicals, light, electric field, and shear stress. Given the substantial variations in pH values observed in chronic wounds, the ability of the hydrogel to modulate pH levels becomes crucial. Consequently, it leads to alterations in ionic strength within a swollen or contracted wound when exposed to higher pH values. This pH-modulating capacity of the hydrogel holds promising potential in wound healing applications, where maintaining an optimal pH environment is vital for promoting effective wound healing processes.

### 3.4. Biological evaluation of porous wound dressing

**Fig 4:**
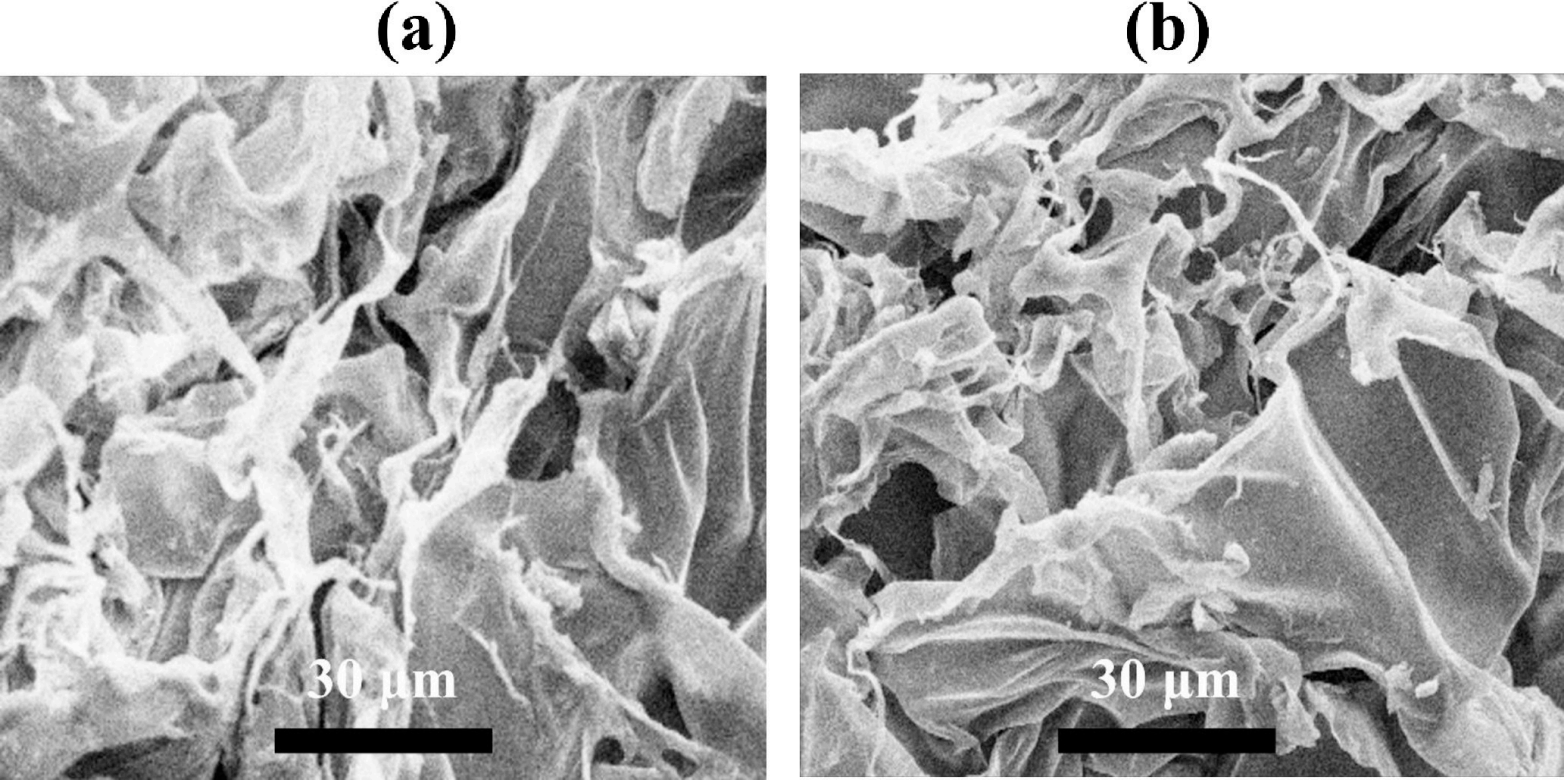
SEM images of prepared wound dressing made of (a) GN-Alg-AgNP, and (b) GN-CH-AgNP after immersion in PBS for 28 days at 25°C.

Fig 4 SEM images show the structural changes of wound dressings immersed in PBS for 28 days. Immersion causes the disappearance of cavities and porosities, leading to chitosan agglomeration due to strong hydrogen bonds. The wound structure consists of irregular interconnected pores (140–250 micrometers) composed of gelatin, AgNP, chitosan, and alginate. SEM images reveal the microstructure of gelatin, chitosan, and alginate nanoparticles. Alginate particles are smaller, and their dispersion is less pronounced. Incorporating nanoparticles enhances mechanical properties and stability.

**Fig 5:**
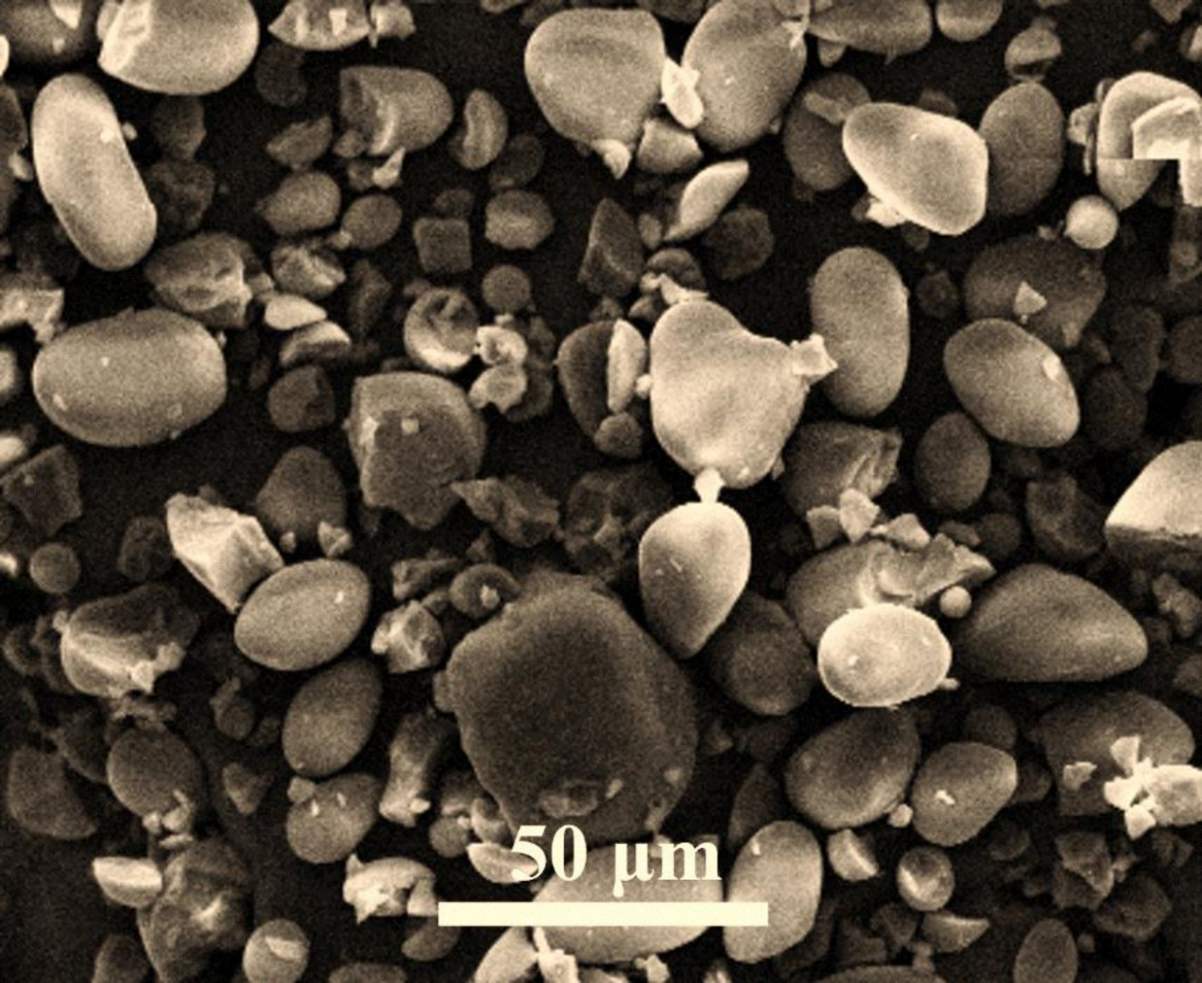
SEM images of pure alginate mixed with chitosan nanoparticles.

The degradation rates for samples S1, S2, S3, and S4 are 37.5%, 39.6%, 44.5%, and 46.5%, respectively, providing insights into material breakdown. Fig 5 presents the SEM image showcasing the polymer composition of chitosan and alginate. The SEM image provides clear evidence of the discernible morphological differences between the two types of nanoparticles. Specifically, the alginate nanoparticles exhibit a spherical shape, while the chitosan nanoparticles display an irregular structure with smooth surfaces. Furthermore, the nanoparticles are observed to be interconnected in the form of agglomerates.

The composite material underwent a homogenization process to achieve a heterogenous combination, aiming to investigate the influence of the nanoparticles’ primary structure on the formation of chemical bonds within the coating structure. Notably, the image reveals that the particle size falls within the range of 25–40 microns, providing valuable information regarding the size distribution and scale of the nanoparticles.

### 3.5. Tensile strength of the porous wound dressing

**Fig 6:**
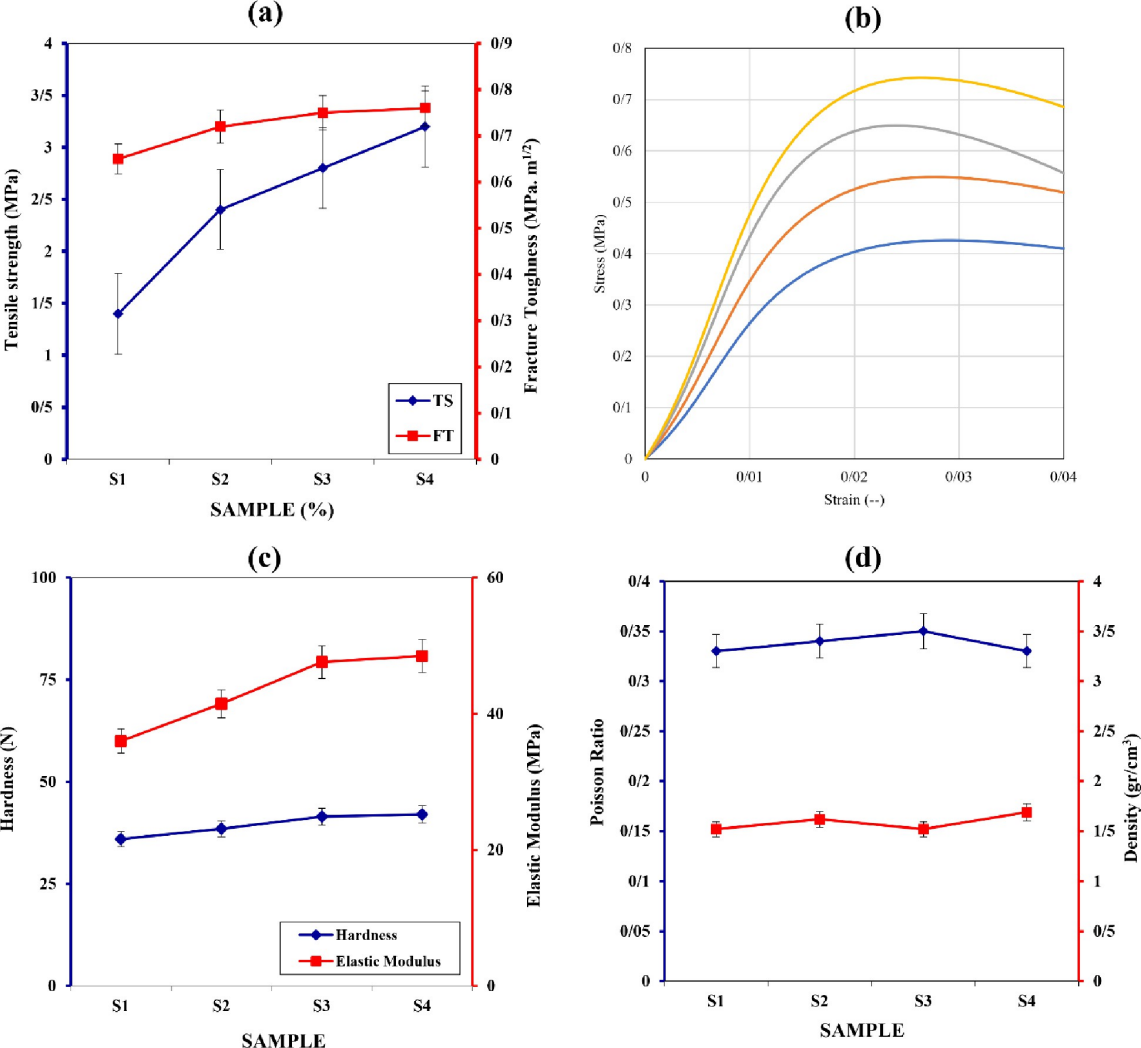
a) Tensile strength vs. fracture toughness, b) stress-strain, c) hardness and elastic modulus, and d) Poisson ratio and density of porous bio-nanocomposites sample S1, S2, S3 and S4.

The addition of silver oxide nanoparticles increases the tensile strength of the prototype from 1.4 MPa to 3.2 MPa, except for sample 3, which decreases to 2.8 MPa. The pure gelatin sample has a tensile strength of approximately 2.4 MPa. The first sample exhibits a porosity change of approximately 51% compared to pure gelatin, and the fracture toughness shows an increasing trend. The presence of chitosan, known for its strong hydrogen bonding, enhances the yield stress of the composite material. The incorporation of sodium alginate nanoparticles decreases the yield stress. Silver oxide nanoparticles are widely used in the medical industry due to their strength, corrosion resistance, fatigue resistance, and crack growth properties. The study provides an overview of the tensile strength, toughness modulus, and physical characteristics of four different wound dressings. Silver oxide nanoparticles have proven to be effective antimicrobial agents in medical and textile applications. The hardness and elastic modulus vary among the samples due to the presence of different components. The addition of silver oxide and chitosan to gelatin does not significantly affect the structure of the samples. The density of the samples exhibits an opposite trend to Poisson’s ratio, with alginate increasing density and chitosan-silver oxide and silver oxide decreasing density. An increase in Poisson’s ratio suggests increased plastic behavior and decreased brittleness, which can be advantageous for wound dressings as shown in Fig 6.

### 3.6. Wettability evaluation

**Fig 7:**
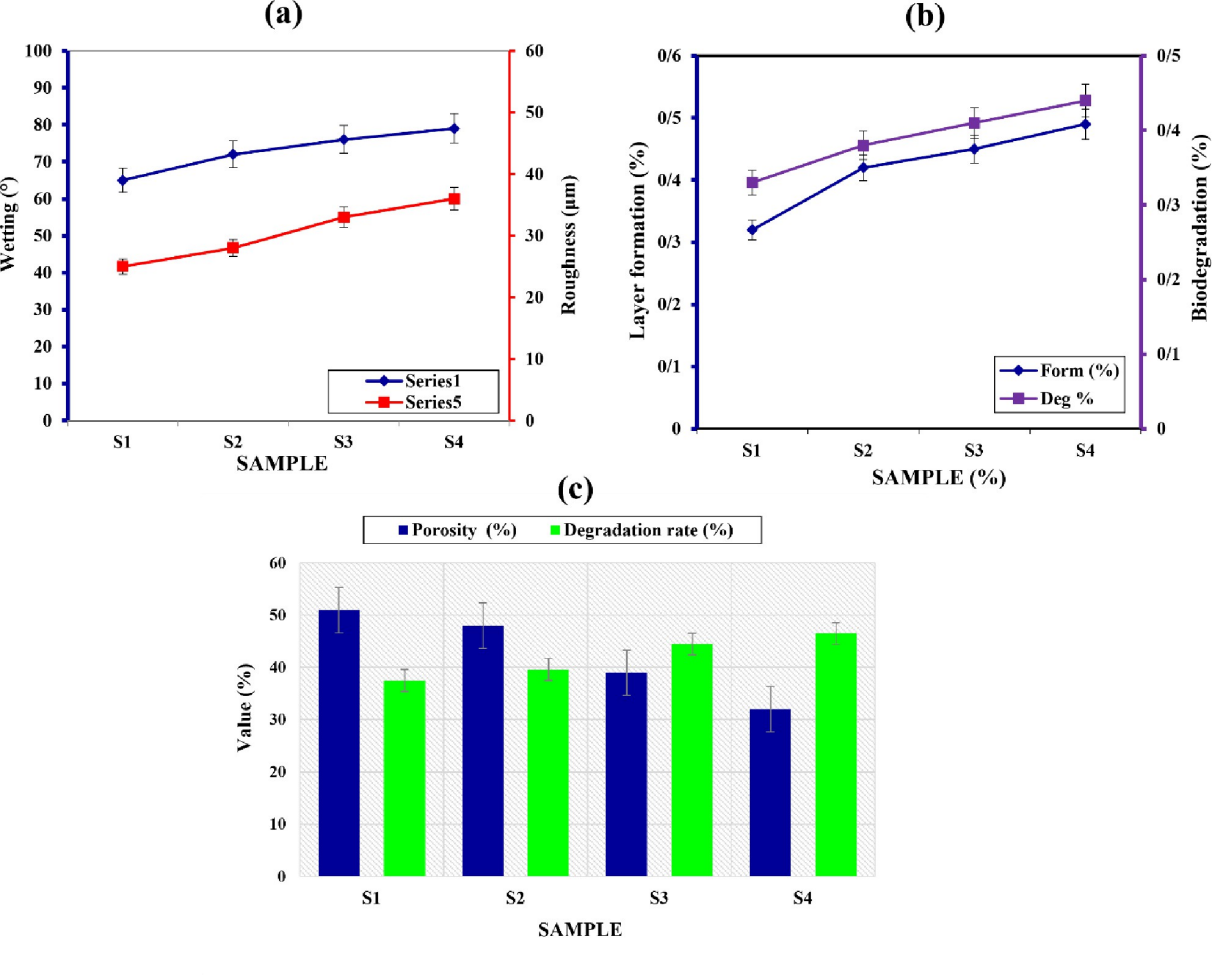
a) Wettability vs. roughness, b) layer formation vs. biodegradation rate, and c) porosity percentages vs. degradation rate of porous nanocomposite wound dressing prepared by FD technique immersed in the PBS solution after 1 day at 37°C.

Fig 7(B) provides insights into the degradation rates of the samples (S1, S2, S3, and S4) after immersion in a PBS solution at 37°C for 1 day. The revised degradation rates, expressed as percentages, are as follows: 37.5% for sample 1, 39.6% for sample 2, 44.5% for sample 3, and 46.5% for sample 4. These values indicate a significant level of degradation within the analyzed period, with higher degradation rates observed in samples with higher percentages. The presence of silver oxide nanoparticles in the samples accelerates the degradation process. It is worth noting that the degradation rates may vary depending on the specific conditions and experimental setup used. Further studies are required to fully understand the implications and potential applications of these degradation rates in the context of wound dressings.

The figure denoted as Fig 7(A) illustrates the interrelationship among porosity, wettability, and roughness of porous nanocomposite wound dressings fabricated using the FD technique. The porosity values, expressed as percentages, progressively decrease from sample 1 to sample 4: 51 ± 5% for sample 1, 48 ± 5% for sample 2, 39 ± 5% for sample 3, and 32 ± 5% for sample 4. Conversely, the wettability, indicated by the contact angle, increases with each subsequent sample: 65° for sample 1, 72° for sample 2, 76° for sample 3, and 79° for sample 4. Correspondingly, the roughness of the dressings also increases from sample 1 to sample 4, with roughness values of 25 μm, 28 μm, 33 μm, and 36 μm, respectively. These findings imply that as the porosity decreases and roughness increases, the wettability of the dressings improves, suggesting a decline in their moisture absorption and retention capabilities. Fig 7(B) focuses on the degradation rates of the samples (S1, S2, S3, and S4) after immersing them in a PBS solution at 37°C for 1 day. The degradation rates, expressed as percentages, are as follows: 37.5% for sample 1, 39.6% for sample 2, 44.5% for sample 3, and 46.5% for sample 4. These values signify significant degradation within the analyzed period, with higher rates observed in samples with higher percentages. Notably, the presence of silver oxide nanoparticles in the samples accelerates the degradation process. In Fig 7(C), a comparison is drawn between the porosity percentages and the degradation rates of the samples. The results demonstrate a correlation between higher porosity and lower degradation rates. Fig 7A–7C indicates that the porous nanocomposite wound dressings undergo alterations in porosity, wettability, roughness, and degradation rates. Furthermore, the inclusion of silver oxide nanoparticles influences properties such as tensile strength, elastic modulus, and degradation rate in the dressings. Further research is imperative to comprehensively comprehend the implications of these findings and explore the potential clinical applications of these wound dressings.

### 3.7. Antibacterial behavior

**Fig 8:**
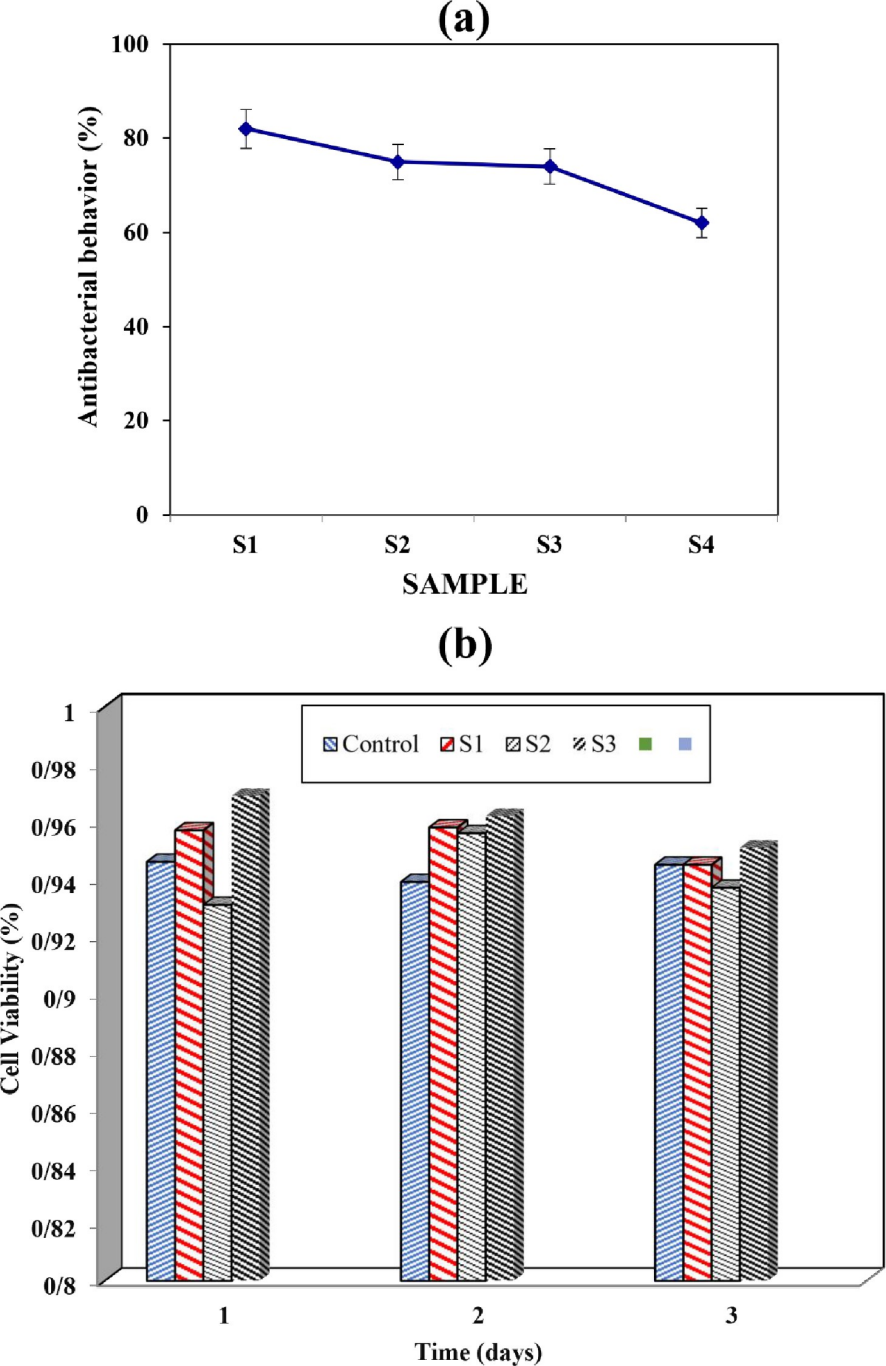
a) Antibacterial behavior of S. aureus and E. coli pathogens halo and antibacterial bar chart for 4 various sample and b) cell analysis after 3 days.

Fig 8 presents a comprehensive analysis of the results obtained, focusing on the porosity, polymer growth rate, and weight loss of the samples manufactured using the FD technique.

The chart reveals notable differences in antibacterial behavior among the samples, with Sample 1 exhibiting the highest antibacterial efficacy at 82%. Subsequent samples, namely Sample 2, Sample 3, and Sample 4, demonstrate decreasing levels of antibacterial behavior, with values of 75%, 74%, and 62%, respectively. Additionally, the control group initially displayed larger wound sizes compared to the chitosan-treated group. However, after a period of nine days, the wound size in the chitosan-treated group significantly improved, indicating the wound-healing properties of chitosan. Based on the analysis of the collected data, the most effective wound dress was selected, and a smart wound dress was designed by activating the chosen sensors and verifying their accuracy. Physical sensors, such as resistance and thermal sensors, are commonly used in biomedicine to measure parameters related to emergent phenomena and metabolic activity, despite lacking the specificity of biochemical sensors. Biosensors offer real-time benefits in monitoring exudate levels, bacterial concentrations, and tissue regeneration, but integrating impedance devices or pressure sensors with a dressing may pose challenges that require further research. In the context of chronic wounds resulting from burns, diabetes, and other medical conditions, the natural healing process of the skin is often impeded, potentially leading to persistent infection and the possibility of amputation. This study incorporated humidity and temperature sensors to facilitate the control and monitoring of infections and inflammation in the samples. It was observed that the presence of blood in the wound area and platelet consumption could contribute to delayed wound healing, while hemolytic anemia, characterized by specific blood parameters and the absence of certain symptoms, may hinder wound healing.

### 3.8 Protein mechanism

**Fig 9:**
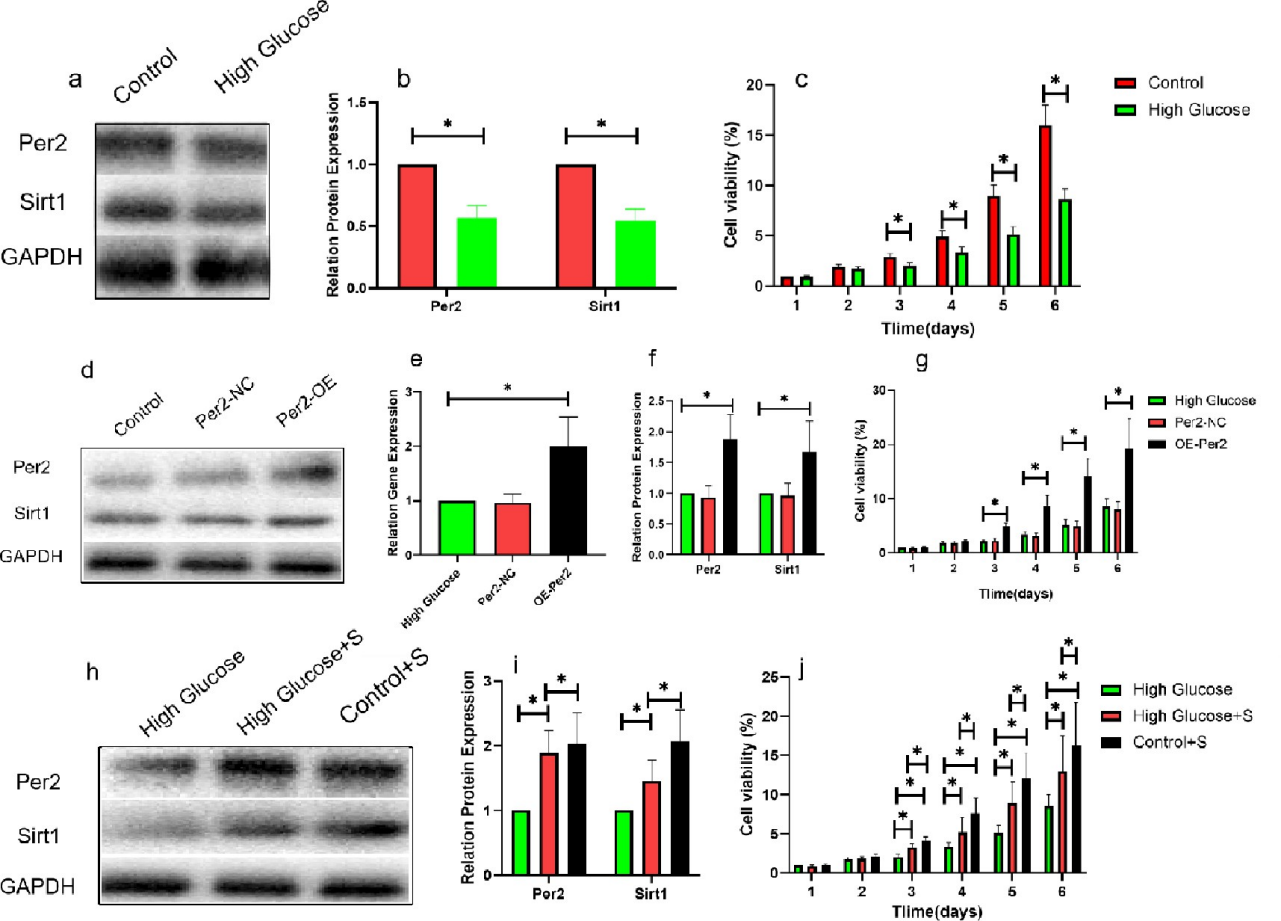
High sugar treatment (a/b) Per2 and Sirt1 protein expression, (c) MTT values; Per2 gene overexpression (d/f) Per2, Sirt1 protein expression, (e) Per2 gene expression. (g) MTT values; material treatment: (h/i) Per2, Sirt1 protein expression; (j) MTT values.

Fig 9. On the sixth day after treatment with 50 mM glucose, the protein expression of Per2 and Sirt1 in Human Umbilical Vein Endothelial Cells (HUVECs) cells decreased, and the cell proliferation activity decreased accordingly; however, after overexpression of Per2 gene, the expression of Per2 protein in cells was up-regulated, while the expression of Sirt1 protein and the cell proliferation activity were partially up-regulated, which indicated that the decrease of cell proliferation activity in the treatment with high glucose might be caused by the down-regulation of Per2 expression and the further down-regulation of Sirt1 expression.

**Fig 10:**
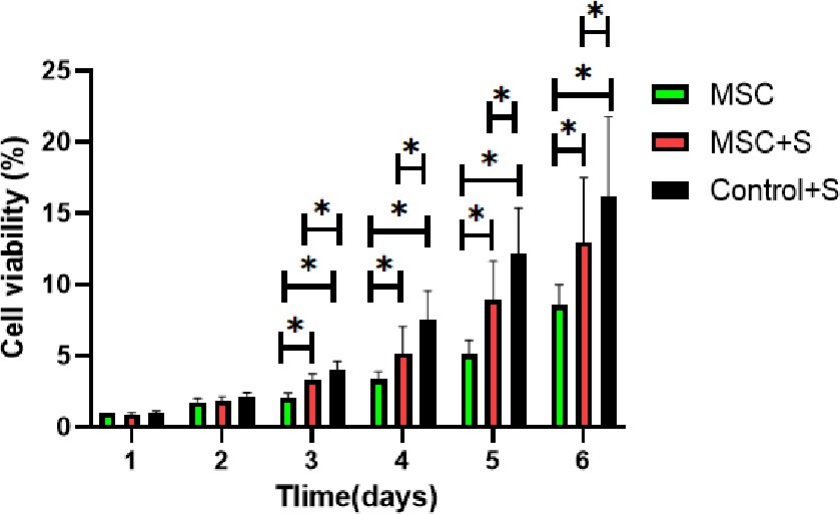
MTT was used to detect the activity of mesenchymal stem cells co-cultured with materials.

Fig 10. MTT was used to detect the activity of mesenchymal stem cells co-cultured with materials, suggestting that the material was not toxic when cultured with stem cells. This suggests that the decrease of cell proliferation activity in high sugar-treated cells may be caused by the down-regulation of Per2 expression and the further down-regulation of Sirt1 expression, whereas after the treatment of cells with the material, the expression of Per2 and Sirt1 proteins was partially regulated, and the proliferation activity of cells was partially restored, which suggests that the mechanism of this material’s cell-proliferative effect is related to the up-regulation of Per2 and further mediated by the up-regulation of Sirt1.

## 4. Conclusion

In this study, novel biocomposite nanocomposite wound dressings were developed and evaluated for their antimicrobial capacity and potential to promote the repair of high glucose-induced vascular injury. Gelatin, chitosan and alginate polymers were combined with silver oxide nanoparticles using green freeze-drying method. Characterization results showed that the nanoparticles formed were approximately 150 nm in size and had an amorphous structure. The dressing has a porosity of 40–60% and a pore size of 140–250 μm, which is favorable for cellular activity. Mechanical tests showed that the chitosan dressing containing silver oxide nanoparticles had a tensile strength of 3.2 MPa and a modulus of elasticity of 48.5 MPa, which showed improved performance compared to other formulations. Notably, the gelatin-silver oxide nanodressing exhibited the most effective antimicrobial activity against the common wound pathogens Staphylococcus aureus and Escherichia coli. In addition, the chitosan-arginine dressing controlled the release of nitric oxide, which has been validated by chemical experiments. Nitric oxide has potent vasodilatory and angiogenic effects, favoring the regeneration of damaged blood vessels. CS-AL dressings also show optimal absorbency and degradation properties, making them suitable for use as wound dressings. The physical and mechanical properties of the developed dressings are enhanced by the incorporation of silver oxide nanoparticles. These multifunctional wound dressings have the potential to promote microvascular regeneration and antimicrobial capacity and further address the clinical challenge of diabetic wound complications. Finally, it was found that the molecular mechanism in which the material is involved is related to PER2-mediated upregulation of SIRT1 expression.
